# Global age-structured spatial modeling for emerging infectious diseases like COVID-19

**DOI:** 10.1093/pnasnexus/pgad127

**Published:** 2023-04-25

**Authors:** Yixiong Xiao, Jingbo Zhou, Qu Cheng, Jun Yang, Bin Chen, Tao Zhang, Lei Xu, Bo Xu, Zhehao Ren, Zhaoyang Liu, Chong Shen, Che Wang, Han Liu, Xiaoting Li, Ruiyun Li, Le Yu, Dabo Guan, Wusheng Zhang, Jie Wang, Lin Hou, Ke Deng, Yuqi Bai, Bing Xu, Dejing Dou, Peng Gong

**Affiliations:** Business Intelligence Lab, Baidu Research, Beijing 100193, China; Business Intelligence Lab, Baidu Research, Beijing 100193, China; Department of Epidemiology and Biostatistics, School of Public Health, Tongji Medical College, Huazhong University of Science and Technology, Wuhan 430074, China; Ministry of Education Key Laboratory for Earth System Modeling, Department of Earth System Science, Tsinghua University, Beijing 100084, China; Division of Landscape Architecture, The University of Hong Kong, Hong Kong 999007, China; Ministry of Education Key Laboratory for Earth System Modeling, Department of Earth System Science, Tsinghua University, Beijing 100084, China; Vanke School of Public Health, Tsinghua University, Beijing 100084, China; Ministry of Education Key Laboratory for Earth System Modeling, Department of Earth System Science, Tsinghua University, Beijing 100084, China; Ministry of Education Key Laboratory for Earth System Modeling, Department of Earth System Science, Tsinghua University, Beijing 100084, China; Center for Statistical Science, Tsinghua University, Beijing 100084, China; Department of Industrial Engineering, Tsinghua University, Beijing 100084, China; Center for Statistical Science, Tsinghua University, Beijing 100084, China; Department of Industrial Engineering, Tsinghua University, Beijing 100084, China; Center for Statistical Science, Tsinghua University, Beijing 100084, China; Department of Industrial Engineering, Tsinghua University, Beijing 100084, China; Business Intelligence Lab, Baidu Research, Beijing 100193, China; Ministry of Education Key Laboratory for Earth System Modeling, Department of Earth System Science, Tsinghua University, Beijing 100084, China; School of Public Health (SPH), Nanjing Medical University, Nanjing 211166, China; Ministry of Education Key Laboratory for Earth System Modeling, Department of Earth System Science, Tsinghua University, Beijing 100084, China; Ministry of Education Key Laboratory for Earth System Modeling, Department of Earth System Science, Tsinghua University, Beijing 100084, China; Department of Computer Science and Technology, Institute of High Performance Computing, Tsinghua University, Beijing 100084, China; State Key Laboratory of Remote Sensing Science, Aerospace Information Research Institute, Chinese Academy of Sciences, Beijing 100101, China; AI for Earth Laboratory, Cross-Strait Institute, Tsinghua University, Beijing 100084, China; Center for Statistical Science, Tsinghua University, Beijing 100084, China; Department of Industrial Engineering, Tsinghua University, Beijing 100084, China; Center for Statistical Science, Tsinghua University, Beijing 100084, China; Department of Industrial Engineering, Tsinghua University, Beijing 100084, China; Ministry of Education Key Laboratory for Earth System Modeling, Department of Earth System Science, Tsinghua University, Beijing 100084, China; Ministry of Education Key Laboratory for Earth System Modeling, Department of Earth System Science, Tsinghua University, Beijing 100084, China; Business Intelligence Lab, Baidu Research, Beijing 100193, China; Department of Geography and Department of Earth Science, The University of Hong Kong, Hong Kong 999007, China

**Keywords:** emerging infectious disease, global airline network, COVID-19

## Abstract

Modeling the global dynamics of emerging infectious diseases (EIDs) like COVID-19 can provide important guidance in the preparation and mitigation of pandemic threats. While age-structured transmission models are widely used to simulate the evolution of EIDs, most of these studies focus on the analysis of specific countries and fail to characterize the spatial spread of EIDs across the world. Here, we developed a global pandemic simulator that integrates age-structured disease transmission models across 3,157 cities and explored its usage under several scenarios. We found that without mitigations, EIDs like COVID-19 are highly likely to cause profound global impacts. For pandemics seeded in most cities, the impacts are equally severe by the end of the first year. The result highlights the urgent need for strengthening global infectious disease monitoring capacity to provide early warnings of future outbreaks. Additionally, we found that the global mitigation efforts could be easily hampered if developed countries or countries near the seed origin take no control. The result indicates that successful pandemic mitigations require collective efforts across countries. The role of developed countries is vitally important as their passive responses may significantly impact other countries.

**Significance Statement**

By incorporating global geographic information in age-structured disease transmission models, we build a pandemic simulator to explore the global dynamics of emerging infectious diseases (EIDs) like COVID-19. Our results emphasize the importance of strengthening the global public health surveillance capacity to prevent future outbreaks and the crucial role of developed countries in maintaining the collective mitigation efforts of the world. The findings of this study offer valuable insights into the understanding of the global pandemic evolution process and provide decision support for improving the global pandemic preparedness capability.

## Introduction

The continuing spread of COVID-19 has become the most severe health crisis in the last decades. For emerging infectious diseases (EIDs) like COVID-19, pharmaceutical solutions are typically unavailable at the beginning of the outbreak. Mitigation strategies are thus focused on nonpharmaceutical interventions (NPIs), most of which are social distancing measures like school closure and remote working that are used to suppress local disease transmissions in the population by reducing human contacts in corresponding social settings like schools and workplaces (1–3). Assessing the control effects of NPIs relies on age-structured social contact matrix data like the POLYMOD data (4). The POLYMOD project constructs age-structured social matrix data (i.e. the POLYMOD data) in four social settings: home, school, workplace, and the general community (e.g. bars, shops, and playgrounds) based on contact surveys in 10 countries. Prem et al. (5) further developed a Bayesian hierarchical model and projected the social contact matrix data in 152 countries based on the POLYMOD data. These data are extensively used in age-structured disease transmission models and explore how various social distancing measures could help suppress the pandemic spread in countries like China (2, 6, 7), the United Kingdom (3, 8), and the United States (9). However, most of these studies focused on the analysis of individual countries.

The COVID-19 pandemic, marked by its rapid worldwide spread and profound global impact, is the most serious public health threat since the 1918 H1N1 influenza pandemic. One of the lessons from COVID-19 is to recognize the possibility of a pandemic starting from anywhere on earth and quickly spreading across the world. Incorporating global geographic information could play a critical role in the study of EIDs. Meanwhile, the spatial couplings of the pandemic spread across different countries determine that the NPIs taken in one country could influence the pandemic evolution in other countries. Studies of EIDs should focus not only on their local transmissions in different social settings but also on the pandemic spread across global geographic regions. However, much of the published research on global pandemic simulations fails to incorporate both aspects together. For example, one type of work mainly focuses on modeling the pandemic spread across regions and examining the effects of travel restrictions (10–12). Another type of work models the global population as one compartment and neglects the spatial heterogeneity of disease transmissions in different regions (13). Modeling frameworks are in need to integrate age-structured disease transmission models across different regions to assess the impacts of EIDs and evaluate the effect of NPIs on the global scale.

The primary objective of this study is to develop a pandemic simulator that accounts for two essential aspects of the global spread of EIDs: (i) age-structured disease transmissions and (ii) the pandemic spread across global regions (Figs. S1 and S2). Based on the simulator, we explored the global pandemic evolutions of EIDs and the effect of control measures under a wide range of scenarios. We explored the dynamics of unmitigated pandemics seeded in different cities to examine the potential risks of infectious diseases that emerged in different cities. We also investigated the effect of different control measures through comparisons with the unmitigated scenario. Finally, we examined the influence of pandemic evolutions between countries and evaluated how much the global mitigation effort could be affected if individual countries take no control.

## Results

### Baseline scenarios

**Fig. 1.**
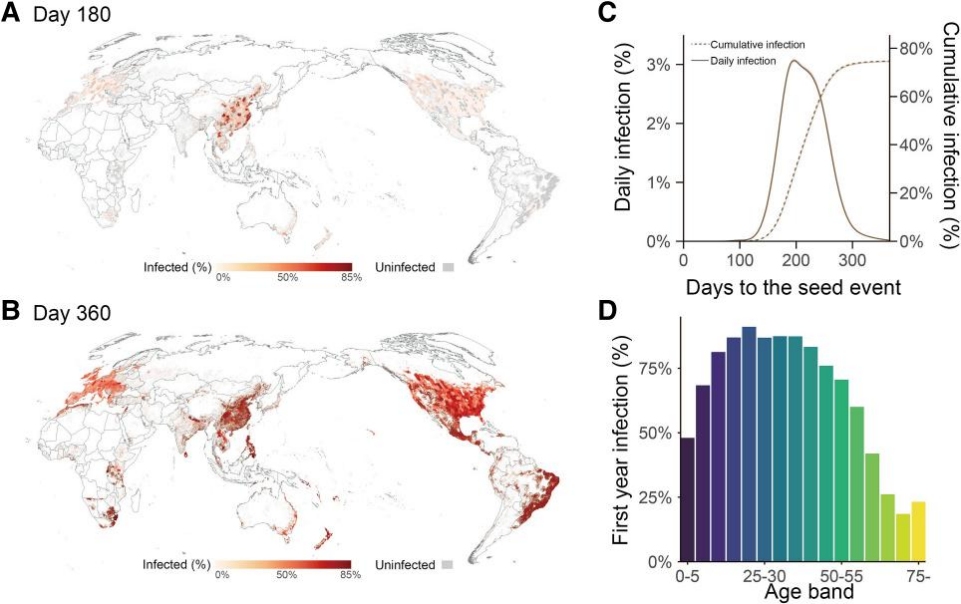
Global pandemic dynamics of the baseline scenario. A, B) Snapshots of the global spread pattern of a single baseline simulation with $R_{0}\;=\;2.4$. Global administrative units are represented by dots with different colors. C) Daily and cumulative infection curves in the baseline scenario. The solid line represents the daily infection curve; the dashed line represents the cumulative infection curve. D) Age-specific infection rate by the end of the first year in the baseline scenario.

We first present the baseline scenario results over 100 simulations under the assumption of no interventions taken to mitigate the pandemic. Figure S1A and B show the expected patterns of the global pandemic spread with $R_{0}=2.4$. The pandemic is first established around the seed origin and then rapidly distributed across the world (Movie S1). The infected population peaks 224 days [95% CI (221, 227)] after the outbreak, with a peak day infection rate of 2.60% [95% CI (2.56%, 2.63%), Fig. 1C]. By the end of the first year, 72.39% [95% CI (72.33%, 72.46%)] of the global population is infected, with 29.99% [95% CI (29.98%, 30.00%)] showing clinical symptoms (Fig. S3). The population between 20 and 55 has higher infection rates than the rest of all age groups (Fig. 1D). The dynamics of the global epidemic spread are sensitive to the virus' transmissibility. Greater $R_{0}$ leads to higher infection rates [$R_{0}$ = 2.7, 77.26%, 95% CI (77.23%, 77.28%)] at the end of the first year and also quicker pandemic evolution processes (Fig. S4 and Movie S1).

**Fig. 2.**
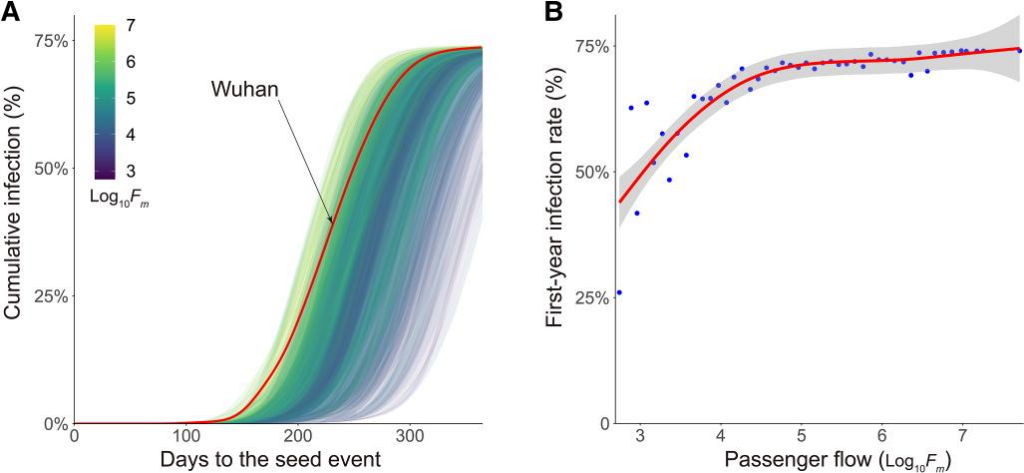
A) The curves of pandemics randomly seeded in 300 cities across the world. Colors indicate the relative size of the seed city's total passenger flow (Log_10_*F*_*m*_). B) The relationship between first-year infection rates and the passenger flow of the seed cities (Log_10_*F*_*m*_). Line denotes the best estimate using GAMs. The gray area denotes the 95% CIs.

We found that the global evolution of the pandemic is dependent on the location of the seed origin. Changing the seed origin would alter the trajectory of the pandemic evolution (Movie S2). Figure 2A shows the curves for pandemics randomly seeded in 300 cities across the world. The color of each curve is determined by the total passenger flow (*F*_*m*_) of the corresponding city. The pandemic evolutions are influenced by the *F*_*m*_ values of the seed city. For example, dense connections of hub cities with large *F*_*m*_ like New York City would facilitate the rapid spread of the pandemic (Movie S2). In contrast, pandemics seeded in cities with small *F*_*m*_ like Gulbarga, India, have slower pandemic processes and lower first-year infection rates (Movie S2). We further used generalized additive models (GAMs) to fit the relationship between the first-year infection rates and *F*_*m*_. As shown in Fig. 2B, the first-year infection rate grows nonlinearly with the increase of *F*_*m*_. For cities with small *F*_*m*_, the first-year infection rate is positively correlated with *F*_*m*_. However, the relationship shows a converging trend for cities with large *F*_*m*_. For cities with Log_10_*F*_*m*_ ≥ 4.5 (over 70% of all cities), the global infection rates are nearly the same at the end of the first year.

### The effects of NPIs

**Fig. 3.**
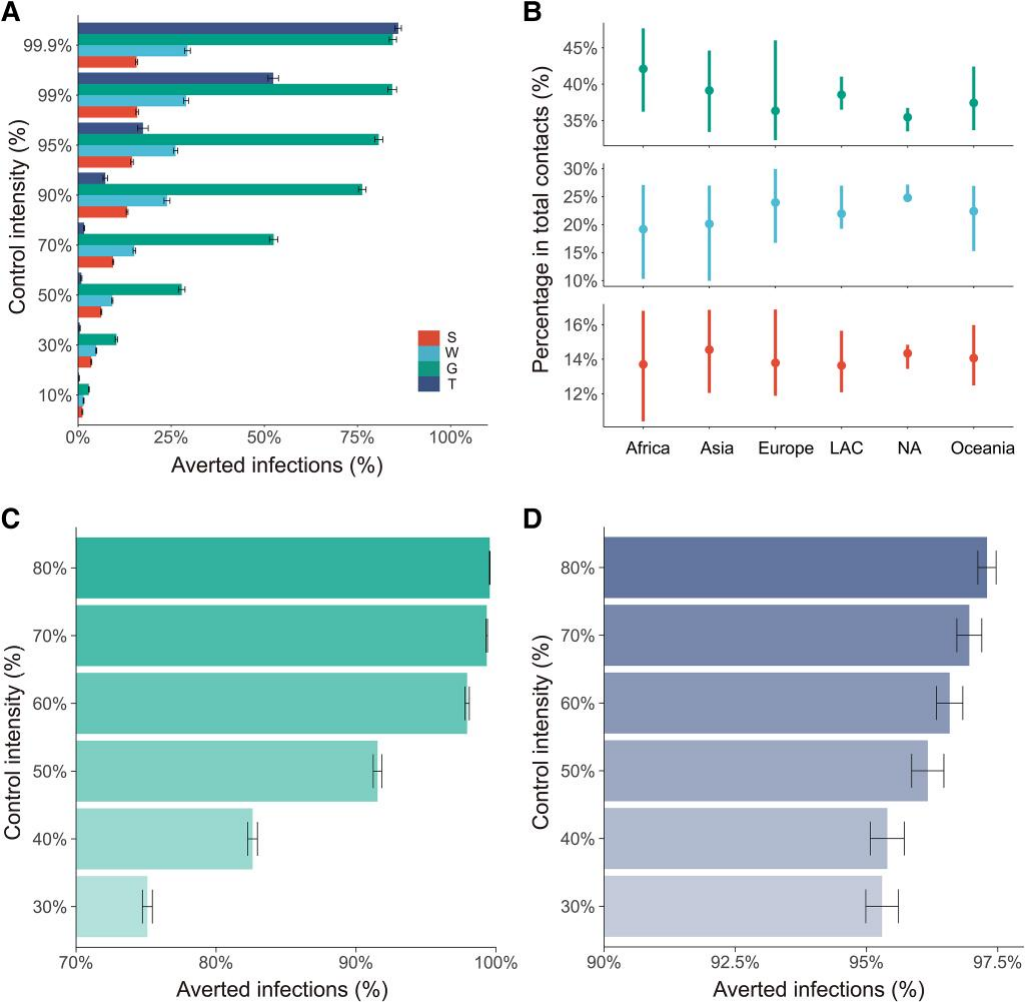
Global effects of NPIs. A) Averted first-year infections of each NPI with different control intensities. S, W, and G indicate the reduction of social contacts in schools, workplaces, and general communities, respectively; T indicates travel restrictions. B) Percentage of contacts in schools (red), workplaces (blue), and general communities (green) summarized in global regions. Dots indicate the mean value in each region; bars indicate the 95% CIs. LAC represents Latin America and the Caribbean region, and NA represents the Northern American region. C) Effects of combined NPIs with school closure (100% school contact reduction), 90% travel restrictions, and different intensities of contact reductions in workplaces and general communities. The *y*-axis denotes contact reductions in workplaces and general communities. D) Effects of combined NPIs with 70% contact reductions in school, workplace, and general communities and different intensities of travel restrictions. The *y*-axis denotes percentages of travel restrictions.

Since the World Health Organization (WHO) declared the COVID-19 pandemic on 2020 March 11, a variety of NPIs have been implemented to contain the pandemic. These NPIs mainly include two groups of controls: social distancing and travel restrictions (14). Social distancing measures aim to suppress disease transmission within cities (1–3), while travel restrictions aim to control the disease spread across cities. We examined the effect of three social distancing measures (i.e. contact reductions in schools, workplaces, and general communities) and travel restrictions by comparing the results of 100 simulations (for each NPI) with the baseline scenario. As experimental controls, each NPI was assumed to be separately implemented 1 week after the identification of the first clinical case. Figure 3A summarizes how much the first-year infection rate would be averted when implementing social distancing measures and travel restrictions with different intensities. It was observed that the relationship between the outcome and the control intensity is different for contact reductions and travel restrictions. Contact reductions in schools (S), workplaces (W), and general communities (G) are consistently effective, as the impacts gradually increase with more intensive controls. In contrast, there is a divergence for the effect of travel restrictions: even though travel restrictions could prevent 85.94% [95% CI (85.10%, 86.79%)] of the global infections with maximum intensity (99.9%), controls with intensities below 90% have almost no effect on the first-year infection rate [lower than 7.31%, 95CI (6.70%, 7.92%)].

The effects of social distancing measures are closely related to the proportion of each contact type. For example, the proportion of contacts in general communities is substantially larger than those in schools and workplaces (Fig. S5). Thus, reducing contacts in general communities is the most effective social distancing measure (Fig. 3A). This correlation implies that governments in different geographic regions could focus on different social distancing strategies based on their contact structure (Fig. 3B). For example, developed regions such as Europe and Northern America have higher labor force participation rates (Fig. 3B). Therefore, people have relatively more contacts at workplaces and fewer contacts in general communities. The situation is the opposite for developing regions like Africa, which have a higher proportion of contacts in general communities and a lower proportion in workplaces (Fig. 3B).

In addition to separate NPIs, we also examined the effects of combined NPIs. Figure 3C presents the averted infections for the combined NPIs of school closure (i.e. 100% school contact reductions), 90% travel restrictions, and different intensities of contact reductions in workplaces and general communities. The results revealed the importance of contact reductions in workplaces and general communities to contain the pandemic spread. Compared with 80% contact reductions in workplaces and general communities which could prevent 99.5% [95% CI (99.5%, 99.5%)] infections, 30% contact reductions in them could only prevent 75.1% [95% CI (74.7%, 75.4%)]. Figure 3D presents the averted infections for combined NPIs of 70% contact reductions in schools, workplaces, and general communities and different intensities of travel restrictions. The result also revealed the importance of travel restrictions as 10% travel restrictions could only prevent 94.0% [95% CI (93.5%, 94.4%)] infections, even combined with 70% social distancing measures.

### Impacts between countries

During the COVID-19 pandemic, global countries have shown discrepancies in their commitment to take actions to suppress disease transmissions. Countries that adopt passive mitigation strategies, like herd immunity (15), might eventually overwhelm their health care system and impact the mitigation efforts of other countries (14). To examine such impacts, we first ran 100 simulations assuming that all countries take the same 120-day control of 70% contact reductions in schools, workplaces, and general communities and 90% travel restrictions 1 week after the first clinical case as a reference of the collective control scenario. In this scenario, 98.18% [95% CI (98.05%, 98.31%)] of global infections would be prevented in the first year. We then chose one country assuming that no NPIs are applied in it (i.e. the NC country) and reran the simulation to monitor its impacts. For each NC country, we ran the simulation 100 times and compared the results with the collective control scenario to test if the infections in the other countries were significantly increased (the significance was tested using two-sample *t* test). Since the impact of the seed origin is incomparable with other countries, we only analyzed the results of nonorigin countries.

**Fig. 4.**
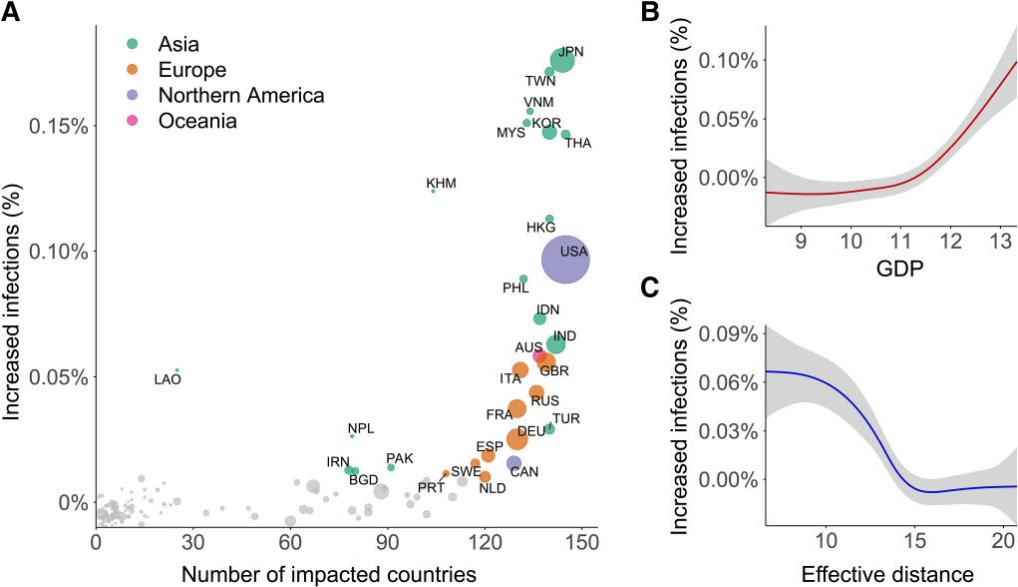
The impact of passive strategies. A) Scatterplot of the number of significantly impacted countries (*x*-axis) and increased infections in the rest of the countries (*y*-axis) in the first year if the NC country takes no control. Dot size indicates the relative size of each country's gross domestic product (GDP) in 2019. Dot color indicates countries in different geographic regions. Gray dots indicate that the global impacts of corresponding countries are not significant at 95% CIs. B) The relationship between increased infections and the GDP of NC country. C) The relationship between increased infections and the effective distance of the NC country to the seed origin. In B) and C), the gray area indicates 95% CIs.

Thirty countries or territories were identified whose passive strategies would significantly increase the infections in the remaining countries (Fig. 4A). Japan has the largest impact which may cause approximately 9.45 million additional infections [0.17%, 95% CI (0.15%, 0.18%)] in other countries. The 30 countries with significant impacts fall into two categories: (i) Asian countries like Japan and Korea which are close to the seed origin and (ii) developed countries in Europe and Northern America like the United Kingdom and the United States. The result implies that the NC country's negative impact is associated with its economic status and proximity to the seed origin. We further used GAMs to analyze the influencing factors of increased infections with two variables: the gross domestic product (GDP) of NC countries and their effective distances from the seed origin (see Materials and methods). As shown in Fig. 4B and C, the relationship between increased infection and GDP presents a nonlinear pattern. The impacts of most countries with low GDP are minor, which presents a flat pattern in low GDP ranges. For countries with high GDP, their impacts significantly increase with the increase in GDP. The relationship between increased infection and the effective distance also shows a nonlinear trend. Countries that are distant from the seed origin show little impact. For countries near the seed origin, their impacts significantly increase with the decrease of effective distance.

Such associations were also observed in the impacts between countries. As shown in Fig. S6, we identified 6,135 impact relations between countries, each of which represents that the infection rate of one country would be significantly increased if another country (i.e. NC country) takes no control. For example, one link from Japan (JPN) to Korea (KOR) means that the infection rate of KOR would be significantly increased if JPN takes no control. As displayed in Fig. S6A, 4,632 (75.50%) of these impact relations are directed from countries with higher GDP to countries with lower GDP, tripling the number from the opposite direction (1,503, 24.50%). Meanwhile, 3,727 (60.75%) of the intercountry impacts are directed from countries that are closer to the seed origin, while the rest 2,408 (39.25%) are from farther countries (Fig. S6B).

## Discussion

In this study, we developed an age-structured pandemic simulator that could model the spatial–temporal spread of EIDs like COVID-19 on the global scale. Prior to this study, the contact matrix data were mostly used in local age-structured models to evaluate the effect of NPIs in individual countries or regions. The simulator we developed was a novel attempt to integrate age-structured disease transmission models across cities in different countries. The baseline scenario results indicate that an unmitigated pandemic is highly likely to spread across the world regardless of where it originated. For over 70% of global cities, the impacts at the end of the first year are nearly the same if no controls are implemented.

The results inform the need for a systematic global framework for the surveillance of EIDs. Prior studies have shown that current public health surveillance systems have uneven global coverages (16) and many countries lack sufficient resources to maintain high-quality and continuous surveillances (17). As we revealed that unmitigated pandemics seeded in most cities have the potential to cause severe global impacts within 1 year, the need is urgent to strengthen and optimize the global public health surveillance system. Moreover, the results also indicate that the mitigation of EIDs like COVID-19 relies on joint efforts across countries. We found that global mitigation efforts could be significantly hampered if developed countries or countries near the seed origin take no control. Existing studies have shown that human mobility contributes to the increase of COVID-19 infections (18). Developed countries have greater human mobility and broad connections with others and thus are more likely to export infected cases to the rest of the world once no mitigations are enforced. We suggest that developed countries should pay more attention to containing the pandemic as they are more likely to impact other countries or regions.

Our study is subject to several limitations. First, the current study mainly considers the effect of NPIs in the first year of the pandemic, in which pharmaceutical approaches are often unavailable for EIDs. Further studies could implement a pharmaceutical intervention module on the basis of the proposed framework to account for its influences. Second, our simulation results are subject to limitations of the data we used. For example, due to the lack of individual-level data like mobile phone trajectories (19, 20), the current model is unable to evaluate the impacts of health care capacity limitation (21), clinical testing (22), contact tracing (23, 24), and case isolation (25). The static social contact matrix data we used are unable to explicitly capture human contacts in specific locations or specific time periods. Therefore, the proposed model could not track the decline of contacts as several studies did using the Mobility Index (26). Future studies could integrate Mobility Index into the current model if the quantitative relations between social contact matrix data proposed in Prem et al. (5) and the Mobility Index are identified. The airline network is unable to characterize human travels with road and railway transport modes and thus might be biased for the simulations in small regions (10, 27, 28). Besides, the global air traffic network we built is undirected because the monthly air traffic data are symmetrical. When examining the effects of travel restrictions, we applied the same level of restrictions on both directions between two regions. Further studies could try to examine more travel restriction policies with the support of more detailed air traffic data. Third, more scenarios could be considered in future studies. In the Results section, we presented a few examples to demonstrate how the model could provide decision support for the global pandemic mitigation. However, the usage of the model is not limited to the scenarios we explored. For example, we only presented the simulation results in the first year since the outbreak. Future studies could examine the simulation results in the following years. When assessing the effects of NPIs, we assumed that each control was implemented separately 1 week after the identification of the first clinical case. Future studies could also examine the effects of combined NPIs or set different response times. Finally, when modeling the effects of NPIs, we do not distinguish each country's ability in implementing the control. Yet, existing research found that the capability of global countries in taking controls significantly influences the outcome. Future studies could further integrate indices like the Global Health Security (GHS) Index (29) into our model to account for such influences.

This study models the global dynamics of EIDs with epidemiological parameters derived from evidence-based studies of COVID-19. Instead of focusing on the ongoing pandemic evolution, we tried to learn from the pandemic and established a framework that could model the global evolution of EIDs under a wide range of scenarios. The framework could be used to model other EIDs with different epidemiological parameters and support the disease intervention in advance. However, a note of caution is that the simulation results are sensitive to the uncertainty of the parameters. For example, the first-year infection rate significantly increases with greater *R*_0_ (Fig. S7A). Figure S7B presents another example, showing that the first-year infection rate is also significantly influenced by the latency period (*Z*). The modeling results are therefore needed to be interpreted with caution. In addition, as our model did not integrate real-time data sources such as Mobility Index or up-to-date flight information, it is more applicable for the scenario analysis of EIDs, rather than monitoring or predicting the ongoing pandemic.

## Materials and methods

### Age-structured pandemic model

The mathematical formula of the model is as follows:


(1) $$\begin{array}{rl} \frac{dS_{m}^{i}}{dt}=\; & -S_{m}^{i}u_{m}^{i}\alpha_{m}(t)\beta \sum_{\;j=1}^{16}\begin{array}{c} C_{m}^{ij}\frac{I_{m}^{j}+(1-q_{m}^{j})I_{m}^{j}}{N_{m}^{j}} \end{array}+\sum_{n\neq m}F_{nm}^{i}\frac{S_{n}^{i}}{N_{n}^{i}-{\mathrm{Ic}}_{n}^{i}}\\ & -\sum_{n\neq m}F_{mn}^{i}\frac{S_{m}^{i}}{N_{m}^{i}-{\mathrm{Ic}}_{m}^{i}}. \end{array}$$

(2) $$\begin{array}{rll} \frac{dE_{m}^{i}}{dt}=\; & S_{m}^{i}u_{m}^{i}\alpha_{m}(t)\beta \sum_{\;j=1}^{16}C_{m}^{ij}\frac{I_{m}^{j}+(1-q_{m}^{j})I_{m}^{j}}{N_{m}^{j}}-\frac{E_{m}^{i}}{Z}+\sum_{n\neq m}F_{nm}^{i}\frac{E_{n}^{i}}{N_{n}^{i}-{\mathrm{Ic}}_{n}^{i}} & -\sum_{n\neq m}F_{mn}^{i}\frac{E_{m}^{i}}{N_{m}^{i}-{\mathrm{Ic}}_{m}^{i}}. \end{array}$$

(3) $$\frac{d{\mathrm{Ic}}_{m}^{i}}{dt}=\frac{r_{m}^{i}E_{m}^{i}}{Z}-\frac{{\mathrm{Ic}}_{m}^{i}}{\mathrm{Dc}}.$$

(4) $$\frac{d{\mathrm{Ia}}_{m}^{i}}{dt}=\frac{(1-r_{m}^{i})E_{m}^{i}}{Z}-\frac{{\mathrm{Ia}}_{m}^{i}}{\mathrm{Da}}+\sum_{n\neq m}F_{nm}^{i}\frac{{\mathrm{Ia}}_{n}^{i}}{N_{n}^{i}-{\mathrm{Ic}}_{n}^{i}}-\sum_{n\neq m}F_{mn}^{i}\frac{{\mathrm{Ia}}_{m}^{i}}{N_{m}^{i}-{\mathrm{Ic}}_{m}^{i}}.$$

(5) $$\frac{dR_{m}^{i}}{dt}=\frac{{\mathrm{Ic}}_{m}^{i}}{\mathrm{Dc}}+\frac{{\mathrm{Ia}}_{m}^{i}}{\mathrm{Da}}.$$

Here, *i* and *j* are the indexes of a 5-year age band *G* (G={[0,5),[5,6),[10,15),…,[70,75),[75,)},i,j=1,2,3…16), while *m* and *n* are the indexes of cities, (m,n=1,2,3…3157). $S_{m}^{i}$, $E_{m}^{i}$, ${\mathrm{Ic}}_{m}^{i}$, ${\mathrm{Ia}}_{m}^{i}$, $R_{m}^{i}$, and $N_{m}^{i}$ are the susceptible, exposed, symptomatic infected, asymptomatic infected, removed, and the total population of age band $G_{i}$ in city *m*, respectively. *Z* is the average latency period. Dc and Da are the average time of symptomatic and asymptomatic infections (see Table S1). $\alpha_{m}(t)$ is the seasonal factor of virus transmission (Eq. S1 and Fig. S3). β is the transmission rate and is derived from $R_{0}$ (Eqs. S2 and S3). $r_{m}^{i}$ represents the proportion of symptomatic infection of age band $G_{i}$ in city *m*. $u_{m}^{i}$ represents the relative susceptibility of age band $G_{i}$ in city *m*. The values of age-specific symptomatic infection rate $r_{m}^{i}$ and susceptibility $u_{m}^{i}$ are derived from past studies (Table S1) (30). *C* is the contact matrix, where $C_{m}^{ij}$ is the daily number of contacts in age band *i* for per capita population in age band *j* in city *m*. We use $q_{m}^{i}$ to represent the case isolation for the symptomatic infected population of age band $G_{i}$ in city *m*, and $q_{m}^{i}$ is set as 1, denoting absolute isolations (i.e. no social contacts for the isolated population). We use the daily number of people ($F_{mn}^{i}$) in the age band $G_{i}$ flying from *m* to *n* to characterize disease transmission across cities. We assume that the symptomatic infected population (${\mathrm{Ic}}_{m}^{i}$) do not travel between cities, while the asymptomatic infected population (${\mathrm{Ia}}_{m}^{i}$) are free to travel. The core model is integrated stochastically with each term on the right-hand side in Eqs. 1–5 determined using a random sample from the Poisson distribution. Simulations are initialized with one case of each age group in the seed city being infected with all populations assumed to be susceptible. The seed time is set as October 15 following existing modeling studies (31). In the modeling analysis, we set *R*_0_ = 2.4 as a representative example following previous work (9, 30) and set *R*_0_ = 2.1 and *R*_0_ = 2.7 for low and high transmissibility scenarios.

The age-structured social contact matrix $C_{m}^{ij}$ is used to denote the number of daily contacts in age band *i* for per capita population in age band *j* in city *m*. As introduced in previous research, $C_{m}^{ij}$ consists of four types of contacts:


(6) $$C_{m}^{ij}=C_{m}^{ij}(H)+C_{m}^{ij}(S)+C_{m}^{ij}(W)+C_{m}^{ij}(G),$$

where $C_{m}^{ij}(H)$, $C_{m}^{ij}(S)$, and $C_{m}^{ij}(W)$ represent contacts at home, schools, and workplaces. $C_{m}^{ij}(G)$ represents the contacts in all other social settings such as restaurants and shopping malls. The effects of social distancing measures are examined through the manipulation of the four contact matrices. For example, to model the effect of 70% reduction of contacts at workplaces, we could multiply $C_{m}^{ij}(W)$ with 1–70% and run the simulation with the adjusted $C_{m}^{ij}$ (i.e. only 30% of $C_{m}^{ij}(W)$ is retained in the adjusted $C_{m}^{ij}$).

### Global simulation network

The global pandemic simulator we proposed is based on three data: the contact matrix data proposed in Prem et al. (5), the global flight data, and the global population data. Figure S1 presents the workflow we used to integrate the three data.

The global airline data are derived from the International Air Transportation Association (IATA) (https://www.iata.org) (11, 12). The IATA data contain the flight route information of 4,418 commercial airports around the world. For each flight route, the origin airport, the destination airport, and the estimated number of monthly passengers between them are recorded. The coverage of the data set is estimated to cover 99% of global commercial air flights (12). In this study, we used the monthly IATA data from 2019 January to 2019 December to avoid the influence of COVID-19 on air traffic. For each flight route, the monthly total volume was equally disaggregated to each day within the corresponding month (e.g. the traffic volume of January 1 equals to the January total divided by 31). Since the monthly air traffic data between two airports are nearly the same for both directions, we also symmetrized the air traffic data following existing work (28) so that the traffic volume from one airport to another equals the opposite direction.

The global population data we used are the Administrative Unit Center Points (AUCP) population data derived from the fourth version of the Gridded Population of the World (GPWv4) collection (https://sedac.ciesin.columbia.edu/data/collection/gpw-v4). The GPWv4 data are available for five time periods: 2000, 2005, 2010, 2015, and 2020. The 2010 data are produced based on the most detailed worldwide Population and Housing Census in 2010. Data for the rest years are interpolated with the 2010 census. The population data are stored in two formats: one raster format and one vector format. In this study, we use the vector data which consist of 13.5 million administrative unit center points as suggested in Wang and Wu (28). In the original data set, the age-structured population data are only available for 2010 and are categorized in 5-year age bands for all ages from 1 to 90. We first reclass the data to match the age band of the contact matrix proposed in Prem et al. (5) and then rescaled the age band population of 2010 to 2020 based on the ratio of each unit's total population in 2020 and 2010.

The AUCP data are integrated with IATA data to determine how many people each airport in IATA data serves (Fig. S1). Specifically, each AUCP is assigned to the closest airport with two constraints: (i) the assigned airport is within the same country as the AUCP, and (ii) the distance between each AUCP and the assigned airport is smaller than 200 km, a cutoff distance introduced by Balcan et al. (12) to characterize the maximum service area of commercial airports. Existing studies noted that the global flight network is densely connected, and thus, it is computationally consuming and unnecessary to include all nodes and edges to characterize the global spread of disease (28). As shown in Fig. S1, we further use the following constraints to trim the network and increase computational efficiency: (i) all cities should be located within the 152 countries that have social contact matrix data; (ii) all routes should have more than 365 passengers in 2019, assuming at least one daily passenger on average; and (iii) all cities should have more than 500 population, a standard proposed by the European Union to identify clusters of human settlement. The result is a simulation network with 3,157 cities (nodes) and 195,934 flight routes (edges), which covers 7.07 billion population in the world (Fig. S2). The characteristics of cities in different geographic regions are summarized in Table S2.

### Effective distance

The effective distance proposed in Brockmann and Helbing (32) is used to characterize the distance between cities. Compared with geographic distance measures, the effective distance is a better approach to characterizing the dynamics of network-driven contagion phenomena. Specifically, the effective distance ($d_{mn}$) from city *n* to a directly connected city *m* is defined as:


(7) $$d_{mn}=1-\log\;P_{mn},$$

where $P_{mn}$ is the fraction of passenger flow from city *n* to city *m*.

For cities with no direct connections, the effective distance $D_{mn}$ is defined as the shortest effective path between two cities:


(8) $$D_{mn}=\underset{\Gamma }{\min}\;\lambda (\Gamma ),$$

where λ(Γ) is the directed length for ordered path $\Gamma =\{n_{1},n_{2},n_{1},\ldots ,n_{L}\}$.

In this study, we analyze the effective distance of countries from the seed origin. The effective distance from city *n* to country *M* is defined as the mean effective distance from *n* to all cities in $M(M=\{m_{1},m_{2},m_{3},\ldots ,m_{k}\})$:


(9) $$D_{Mn}=\frac{\sum_{k=1}^{M}m_{k}}{N_{k}},$$

where $D_{Mn}$ is the effective distance from city *n* to country *M*. $m_{k}$ is the *k*th city in country *M*. $N_{k}$ is the total number of cities in country *M*.

## Supplementary material

Supplementary material is available at *PNAS Nexus* online.

## Supplementary Material

pgad127_Supplementary_Data

## Data Availability

The sources of the global population demographic data and the social contact matrix data used in the study are outlined in the main text and the supplementary materials. Related codes and data used for the analysis in this paper can be found on https://github.com/Ethan-yxx/Global_Pandemic_Sim.
