# Supplementary material for: Global age-structured spatial modeling for emerging infectious diseases like COVID-19
Source: PNAS Nexus. 2023 Apr 25;2(5):pgad127. doi: 10.1093/pnasnexus/pgad127 (PMC10153731; doi:10.1093/pnasnexus/pgad127)
Supplement: pgad127_Supplementary_Data [file pgad127_supplementary_data.zip › PNASNEXUS-PNASNEXUS-2022-01019-s03.docx]

**
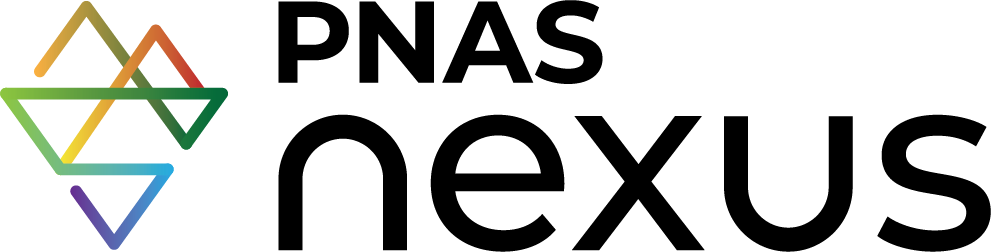
**

**Supplementary Information for**

Global age-structured spatial modeling for emerging infectious diseases (EIDs) like COVID-19

Yixiong Xiao^1^, Jingbo Zhou^1^, Qu Cheng^2^, Jun Yang^3^, Bin Chen^4^, Tao Zhang^3^, Lei Xu^5^, Bo Xu^3^, Zhehao Ren^3^, Zhaoyang Liu^6,7^, Chong Shen^6,7^, Che Wang^6,7^, Han Liu^1^, Xiaoting Li^3^, Ruiyun Li^8^, Le Yu^3^, Dabo Guan^3^, Wusheng Zhang^9^, Jie Wang^10,11^, Lin Hou^6,7^, Ke Deng^6,7^, Yuqi Bai^3^, Bing Xu^3^, Dejing Dou^1^, Peng Gong^12*^

* Peng Gong

**Email:**  penggong@hku.hk

**This PDF file includes:**

Supplementary text

Figures S1 to S6

Tables S1 to S2

Legends for Movies S1 to S2

SI References

**Other supplementary materials for this manuscript include the following:**

Movies S1 to S2

**Supplementary Information Text**

**1. The influence of seasonal forcing**

We use $\alpha_{m}(t)$ to incorporate the seasonal forcing of virus transmission (1–3), as shown in Supplementary Equation (1):

|  | (1) |
| --- | --- |

Here *f* is the influence factor for seasonal effects, *f* = 0 indicates no seasonal effect, *f* = 0.1, 0.2, 0.4, indicate scenarios with low, moderate, and high seasonal effects, respectively. $\phi$ is the phase shift in days, and is set as 15 (corresponding to the date of January 15^th^) as used in Kenah et al., 2011. $\theta_{m}$ is set as 1, 0, -1 for city *m* located in the north of the Northern Tropic, the tropical zone, and south of the Southern Tropic. Determined by S1 Equation 1, cities in the North and South Hemispheres have dual seasonal effects with $\alpha_{m}(t)$ increase in the summertime and decrease in the wintertime, while cities in the tropical zone have constant $\alpha_{m}(t)$ (Fig. S3).

**2. Deriving** $\boldsymbol{\beta}$ **with given** $\boldsymbol{R}_{\mathbf{0}}$

We calculate $\beta$ with $R_{0}$ and the next-generation matrix (NGM). The NGM can be constructed for given infected class $X=[E^{1}\ldots E^{16},{Ic}^{1}\ldots{Ic}^{16},{Ia}^{1}\ldots{Ia}^{16}]$ and uninfected class $Y=[S^{1}\ldots S^{16},R^{1}\ldots R^{16}]$. Since $R_{0}$ is the largest eigenvalue of the NGM, $\beta$ can be reverse-engineered for given NGM and $R_{0}$.

Given the age-specific infected class $X=[E^{1}\ldots E^{16},{Ic}^{1}\ldots{Ic}^{16},{Ia}^{1}\ldots{Ia}^{16}]$ and uninfected class $Y=[S^{1}\ldots S^{16},R^{1}\ldots R^{16}]$, the vectors for new infection ($\mathcal{F}$) and other rates ($\mathcal{v}$) can be formed as shown in SI Equation 2.

|  | (2) |
| --- | --- |

Here, notation follows the nations of Equations 1-5 in the main text. The disease-free equilibrium is $x_{0}=[0,\ldots0,0\ldots0,0,\ldots,0,N,\ldots,N,0,\ldots0]$. The next-generation is constructed as shown in SI Equation 3 (4, 5).

|  | (3) |
| --- | --- |

With the next-generation matrix, $\beta$ can be derived with given $R_{0}$ following the algorithms presented in existing studies (6–8)


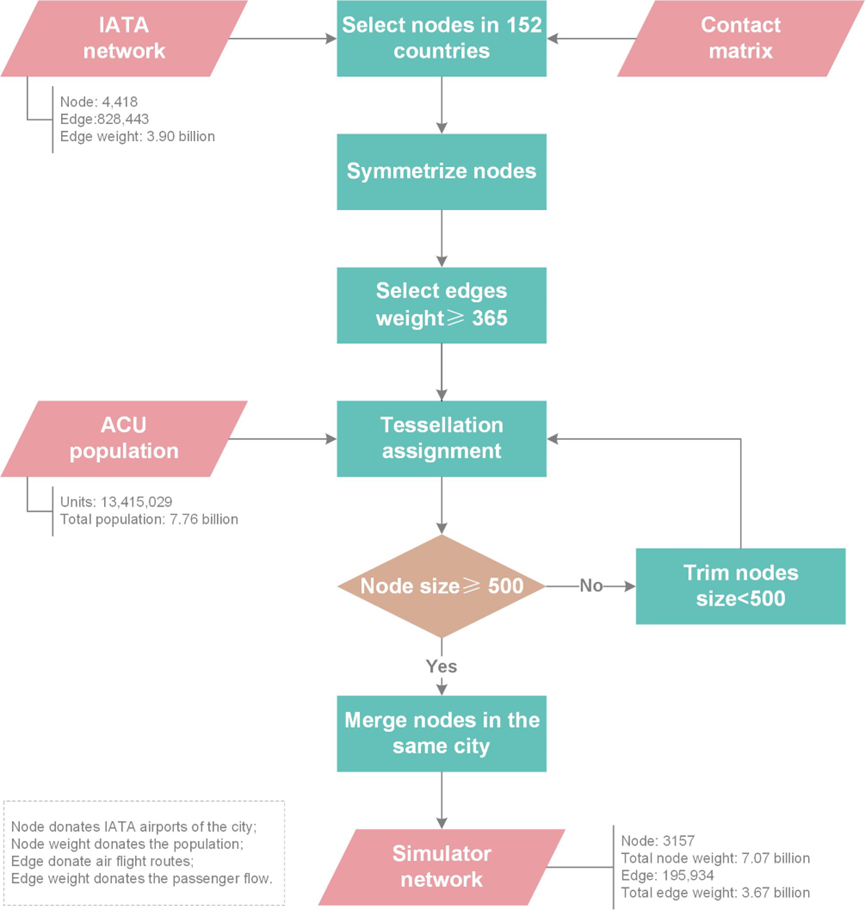


Fig. S1 Workflow for data integration of the pandemic simulator


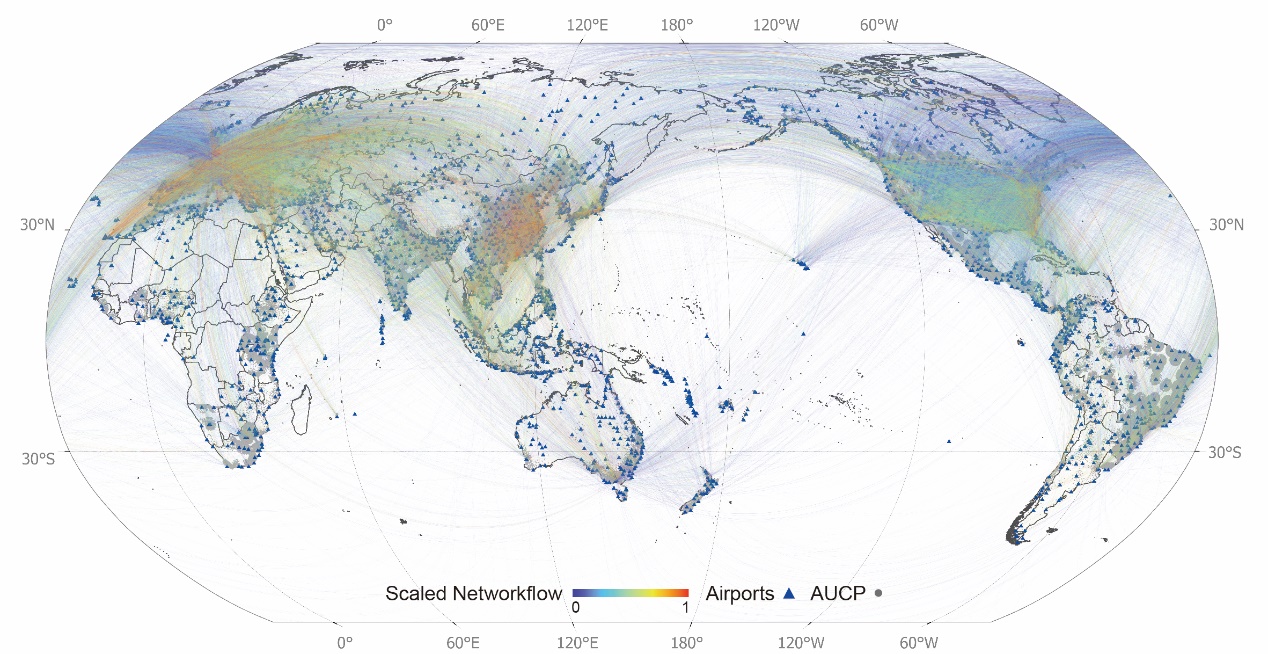


Fig. S2 The network of the global pandemic simulator. Blue triangles denote IATA airports. Grey dots denote AUCPs. Lines denote air flight routes between airports, with the color blue to red representing the relative travel volume from low to high.


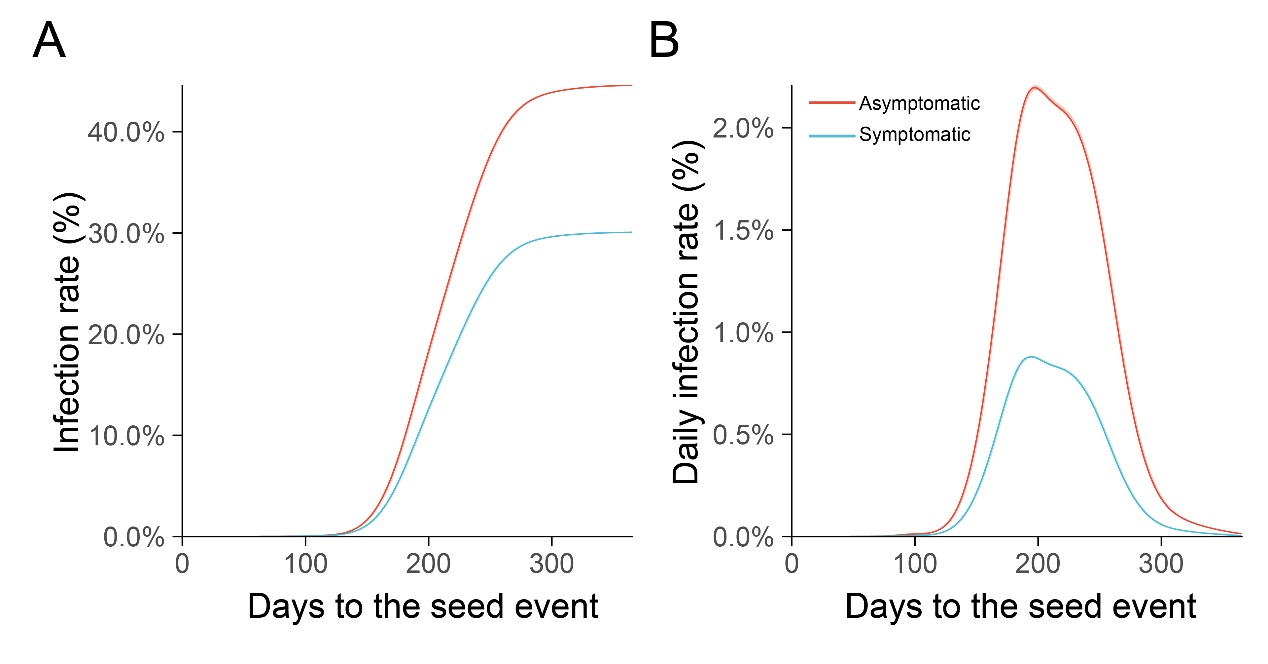


Fig. S3 Demonstration of the symptomatic and asymptomatic infections. (A). The cumulative curve of the symptomatic and asymptomatic infections in the first year. (B) The daily curve of the symptomatic and asymptomatic infections in the first year.


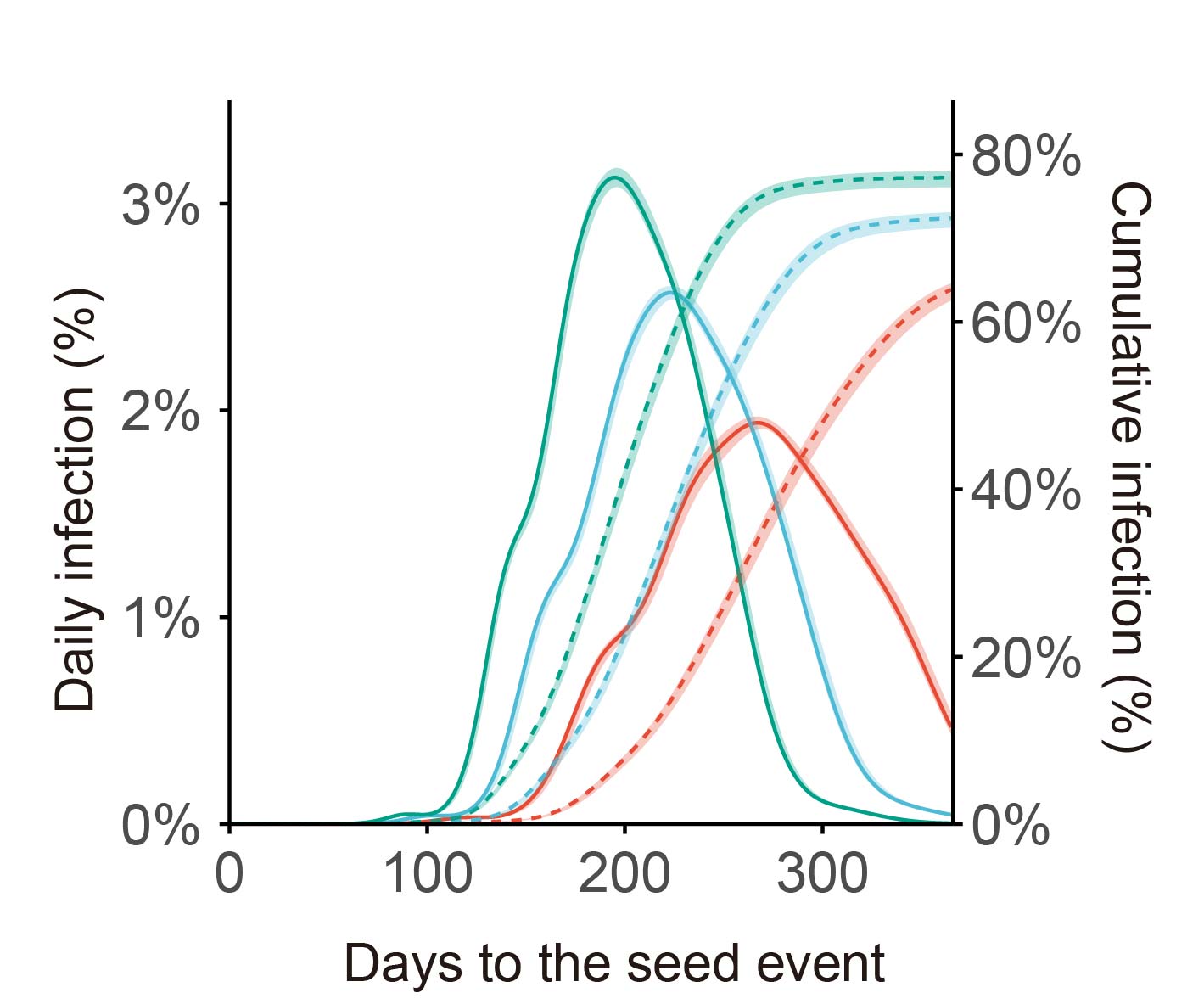


Fig. S4 Daily (solid lines) and cumulative (dashed lines) infection rates with different $\boldsymbol{R}_{\mathbf{0}}$. Red, blue and green colors represent simulations with $\boldsymbol{R}_{\mathbf{0}}\mathbf{=2.1, 2.4, 2.7}$, respectively.


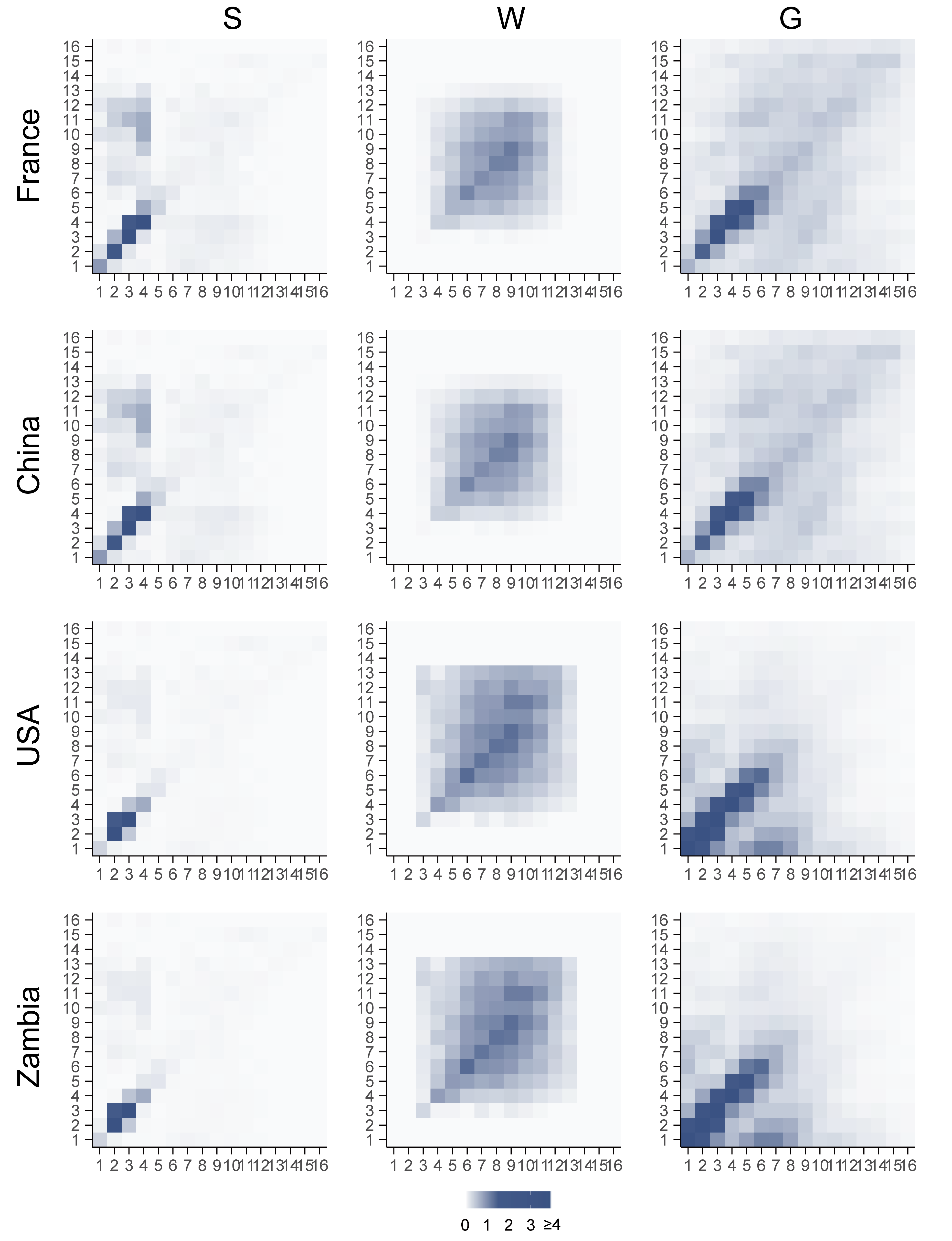


Fig. S5 Age-structure contact patterns in selected countries. Numbers from 1-16 donate age bands of [0-5, 5-10, 10-16,…, 75+)


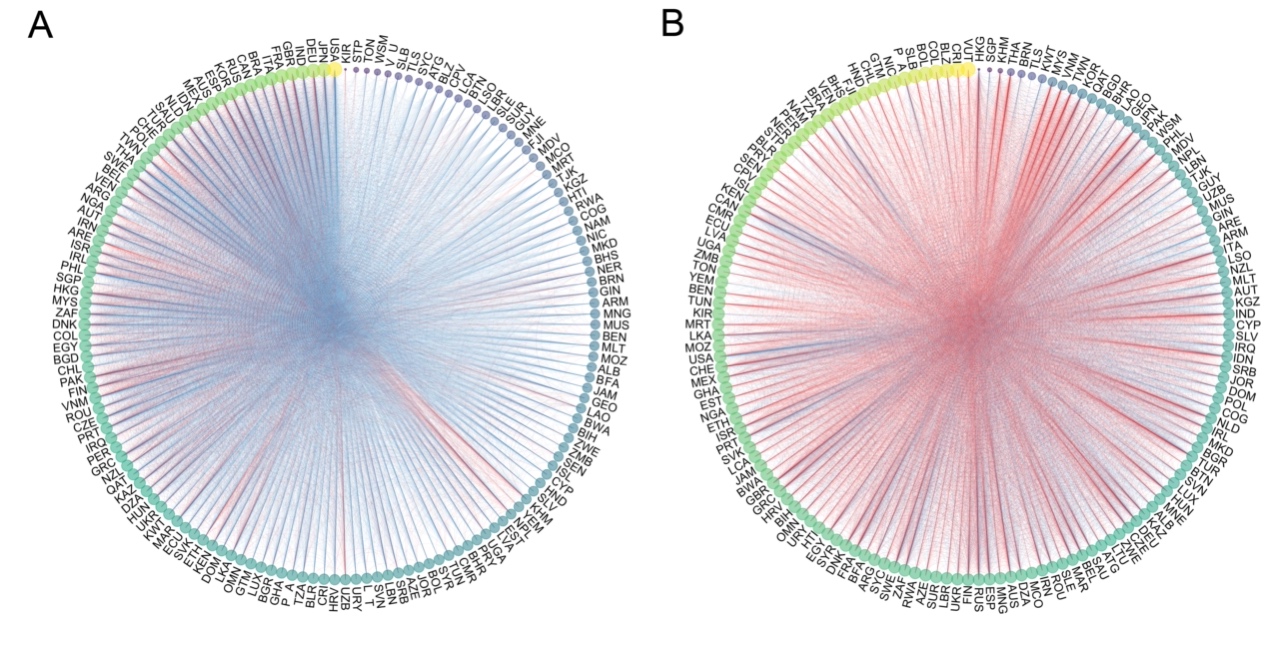


Fig. S6 Impact of the NC country on other countries. (A) Nodes are arranged by the order of GDP. Node size denotes the relative size of GDP. Blue arcs indicate impacts directed from countries with larger GDP while red arcs indicate impacts directed from countries with smaller GDP. (B) Nodes are arranged by the order of effective distances. Node size denotes the relative size of the effective distance. Blue arcs indicate impacts directed from countries with larger effective distances while red arcs indicate impacts directed from countries with smaller effective distances.


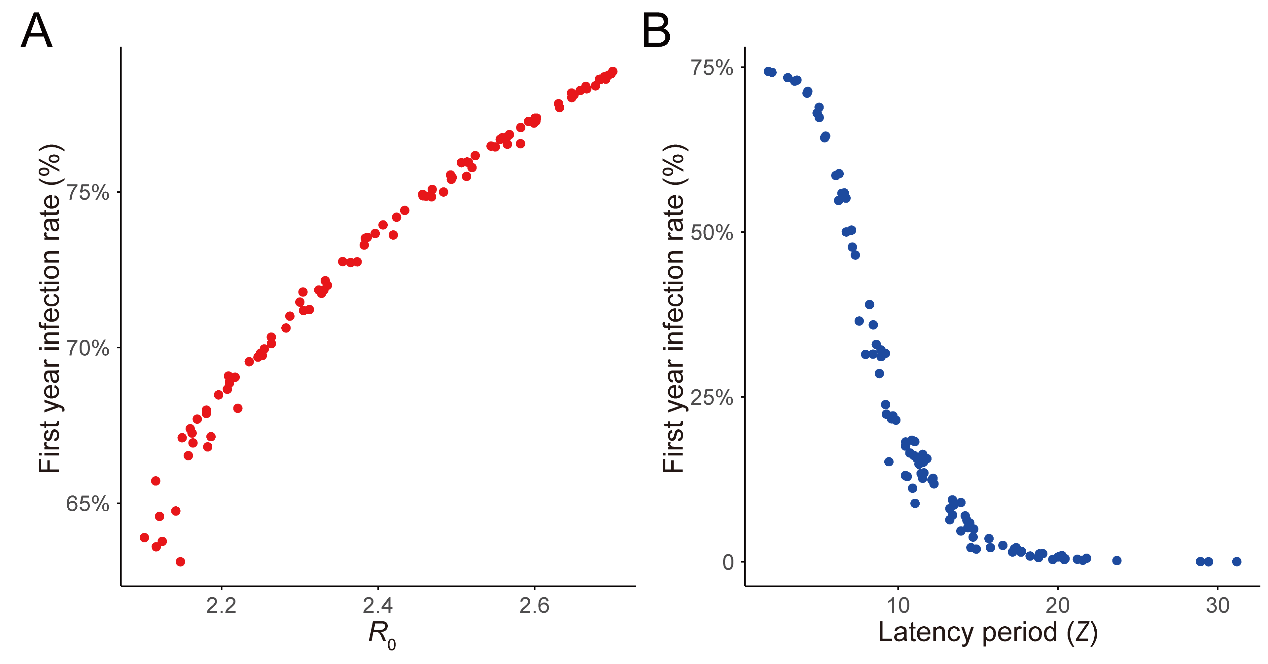


Fig. S7 Firs year infections rates for different
$\text{R}_{\text{0}}$ (A) and latency period *Z* (B)

Table S 1 Parameter values

| Parameter | Value | Reference |
| --- | --- | --- |
| $\text{R}_{\text{0}}$ | 2.1, 2.4, 2.7 | (6, 8, 9) |
| *Z* | 3 | (10, 11) |
| *Dc* | 2.9 | (12) |
| *Da* | 5 | (6, 8) |
| $\text{r}_{\text{m}}^{\text{i}}$ |  | (6, 8) |
| $\text{u}_{\text{m}}^{\text{i}}$ |  | (6, 8) |

Table S 2 Characteristics of Cities

| Region | *N* | ${Pop}_{mean}$ | ${Pop}_{min}$ | ${Pop}_{max}$ |
| --- | --- | --- | --- | --- |
| Africa | 262 | 3495882 | 3327 | 45171517 |
| Asia | 996 | 4436361 | 1250 | 75984635 |
| Europe | 576 | 1215062 | 513 | 19376116 |
| Latin America and the Caribbean | 440 | 1441454 | 858 | 32808982 |
| Northern America | 648 | 571918.5 | 505 | 9736240 |
| Oceania | 235 | 137746.9 | 510 | 5492787 |

Table S 3 Performance of the statistical models

| Model formula | GCV | Deviance explained (%) |
| --- | --- | --- |
| *Infection*=*a_1_*+*f_1_*(*flow*)+*ε*_1_ | 2.5e-03 | 76.4% |
| *Increased_infection*= *a_2_*+*f_2_*(*GDP*)+ *f_3_*(*D_eff_*)+ *ε*_2_ | 6.4e-08 | 56.2% |

*f_1_*(*flow*), *f_2_*(*GDP*) , and *f_3_*(*D_eff_*) are cubic spline smoothing functions for passenger flows (flow), log-transformed GDP (GDP), and effective distance (*D_eff_*), respectively. *a_1_* and *a_2_* are intercepts; *ε*_1_ and *ε*_2_ are error terms for two models.

**Supplementary Movie Captions**

Movie. S1 Global epidemic evolutions with different $\boldsymbol{R}_{\mathbf{0}}$ seeded in Wuhan, China.

Movie. S2 Global epidemic evolutions with different seed origins.

**SI References**

1. S. M. Kissler, C. Tedijanto, E. Goldstein, Y. H. Grad, M. Lipsitch, Projecting the transmission dynamics of SARS-CoV-2 through the postpandemic period. *Science (80-. ).* **368**, 860–868 (2020).

2. B. S. Cooper, R. J. Pitman, W. J. Edmunds, N. J. Gay, Delaying the international spread of pandemic influenza. *PLoS Med.* **3**, 0845–0855 (2006).

3. E. Kenah, D. L. Chao, L. Matrajt, M. E. Halloran, I. M. Longini, The global transmission and control of influenza. *PLoS One* **6** (2011).

4. O. Diekmann, J. A. P. Heesterbeek, J. A. J. Metz, On the definition and the computation of the basic reproduction ratio R0 in models for infectious diseases in heterogeneous populations. *J. Math. Biol.* **28**, 365–382 (1990).

5. O. Diekmann, J. A. P. Heesterbeek, M. G. Roberts, The construction of next-generation matrices for compartmental epidemic models. *J. R. Soc. Interface* **7**, 873–885 (2010).

6. N. G. Davies, *et al.*, Age-dependent effects in the transmission and control of COVID-19 epidemics. *Nat. Med.* (2020) https:/doi.org/10.1038/s41591-020-0962-9.

7. P. G. T. Walker, *et al.*, The impact of COVID-19 and strategies for mitigation and suppression in low- And middle-income countries. *Science (80-. ).* **369**, 413–422 (2020).

8. I. F. Miller, A. D. Becker, B. T. Grenfell, C. J. E. Metcalf, Disease and healthcare burden of COVID-19 in the United States. *Nat. Med.* **26**, 1212–1217 (2020).

9. J. T. Wu, K. Leung, G. M. Leung, Nowcasting and forecasting the potential domestic and international spread of the 2019-nCoV outbreak originating in Wuhan, China: a modelling study. *Lancet* **395**, 689–697 (2020).

10. S. A. Lauer, *et al.*, The Incubation Period of Coronavirus Disease 2019 (COVID-19) From Publicly Reported Confirmed Cases: Estimation and Application. *Ann. Intern. Med.* **172**, 577–582 (2020).

11. J. A. Backer, D. Klinkenberg, J. Wallinga, Incubation period of 2019 novel coronavirus (2019-nCoV) infections among travellers from Wuhan, China, 20-28 January 2020. *Euro Surveill.* **25**, 2000062 (2020).

12. A. J. Kucharski, *et al.*, Early dynamics of transmission and control of COVID-19: a mathematical modelling study. *Lancet Infect. Dis.* **20**, 553–558 (2020).
