# Supplementary figures and images for: Global age-structured spatial modeling for emerging infectious diseases like COVID-19
Source: PNAS Nexus. 2023 Apr 25;2(5):pgad127. doi: 10.1093/pnasnexus/pgad127 (PMC10153731; doi:10.1093/pnasnexus/pgad127)

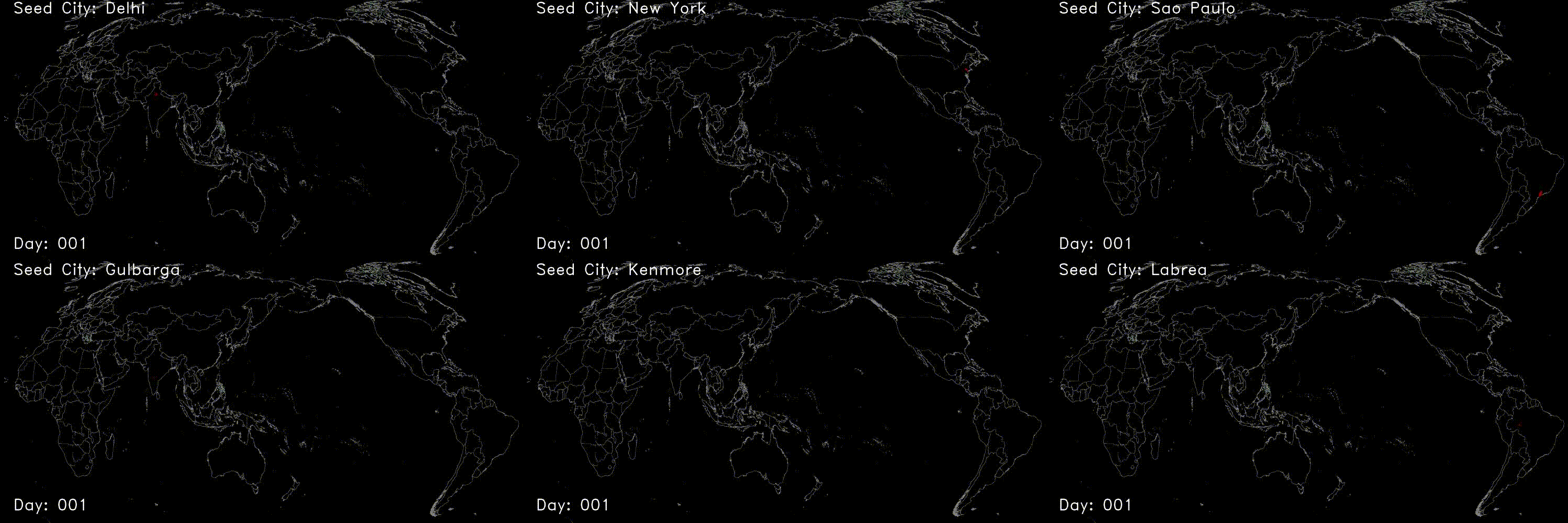

Supplement: pgad127_Supplementary_Data [file pgad127_supplementary_data.zip › PNASNEXUS-PNASNEXUS-2022-01019-s02.gif]

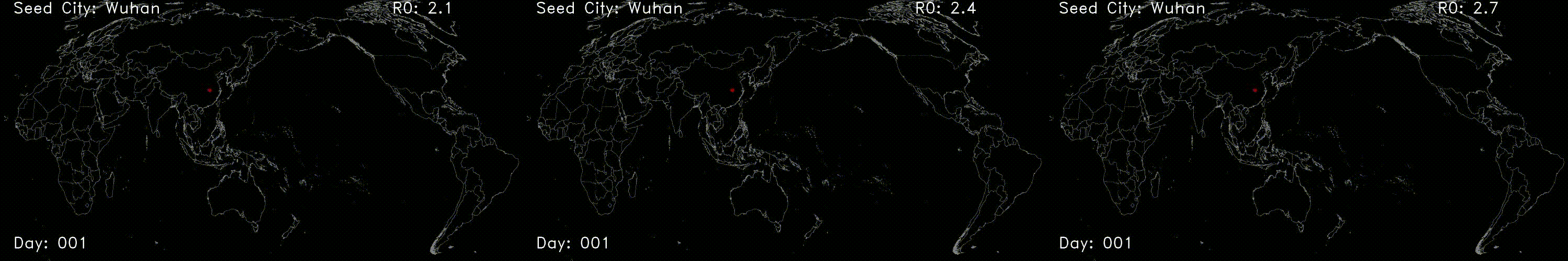

Supplement: pgad127_Supplementary_Data [file pgad127_supplementary_data.zip › PNASNEXUS-PNASNEXUS-2022-01019-s01.gif]
